# 

**Published:** 1837-01-01

**Authors:** 


### CONTENTS

OF THE

### MEDICO-CHIRURGICAL REVIEW,

### No. LI. JANUARY 1, 1837,

### [BEING No. XI. OF A DECENNIAL SERIES.]

### REVIEWS.

I.

The Nature and Treatment of Dropsy considered, especially in reference to the Diseases of the Internal Organs of the Body which most commonly produce it. Parts I. and II. Anasarca and Ascites, &c. By EDWARD J. SEYMOUR, M.D. &c. .. 1

1. Anasarca, or Dropsy of the Skin .. .. .. .. .. .. .. .. .. .. 2

2. Ascites .. .. .. .. .. .. .. .. . .. .. .. .. .. .. 18

II.

The Factory System and Factory Statistics .. .. .. .. .. .. .. .. .. 25

III.

On [illegible] Disease of the Hip-joint. By WILLIAM COULSON, Esq. .. .. .. .. .. 31

IV.

PATHOLOGICAL ANATOMY.

I. Lectures on the Morbid Anatomy of the Serous and Mucous Membranes. By THOMAS HODGKIN, M.D. .. .. .. .. .. .. .. .. .. .. .. .. ..

II. The Clinique Medicale. By G. ANDRAL. Condensed and Translated by D. SPILLAN, M.D. &c. .. .. .. .. .. .. .. .. .. .. .. .. .. .. 40

V.

De la Prostitution de la Ville de Paris. M. DUCHATELET on the Prostitution of Paris. Second and concluding Article .. .. .. .. .. .. .. .. .. 49

VI.

A Treatise on Tetanus, being an Essay for which the Jacksonian Prize was awarded by the College of Physicians. By THOMAS B. CURLING, Esq. .. .. .. .. .. 55

1. General Account of Tetanus .. .. .. .. .. .. .. .. .. .. .. 56

2. Mode of Termination .. .. .. .. .. .. .. .. .. .. .. .. 60

3. Statistics of Tetanus .. .. .. .. .. .. .. .. .. .. .. .. 61

4. Causes of Tetanus .. .. .. .. .. .. .. .. .. .. .. .. 62

5. Pathology of Tetanus .. .. .. .. .. .. .. .. .. .. .. .. 64

6. Treatment .. .. . .. .. .. .. .. .. .. .. .. .. .. 70

VII.

An Account of the most frequented Watering Places on the Continent, &c. By EDWIN LEE, Esq. .. .. .. .. .. .. .. .. .. .. .. .. .. .. .. 84

VIII.

Facts and Cases in Obstetric Medicine, with Observations on some of the most important Diseases of Females. By J. T. INGLEBY, Esq. .. .. .. .. .. .. .. 89

IX.

The Speculum applied to the Diagnostic and Treatment of the Organic Diseases of the Womb. An inaugural Dissertation, &c. By JOHN BALBIRNIE, A.M. .. .. .. 105

X.

Traité sur les Maladies du Foie. Par A. Bonnet .. .. .. .. .. .. .. .. .. 124

XI.

The Fallacy of the Art of Physic, as taught in the Schools: with the Development of new and important Principles of Practice. By Samuel Dickenson, M.D... .. 135

XII.

Homœopathy.

I. A Practical View of Homœopathy; being an Address to British Practitioners on the general Applicability and superior Efficacy of the Homœopathic Method in the Treatment of Disease. By Stephen Simpson, M.D. .. .. .. .. .. ..
II. Animal Magnetism and Homœopathy, &c. By Edwin Lee, M.R.C.S. .. ..
III. Exposition of Homœopathy. By Dr. Bureaud Rioffrey.. .. .. .. .. ..
IV. Articles on Homœopathy in the Medical Gazette, Nos. 467 and 469 .. .. .. } 137

### PERISCOPE.

### Bibliographical Notices.

1. Retrospective Address, delivered at the Fourth Anniversary Meeting of the Provincial Association. By John Green Crosse, Esq. .. .. .. .. .. .. .. .. 145
  1. Medical Statistics of the Army .. .. .. .. .. .. .. .. .. 146
  2. Retraction of the Tongue .. .. .. .. .. .. .. .. .. .. 146
  3. Entozoa in the Human Body. .. .. .. .. .. .. .. .. .. 147
  4. Homœopathy .. .. .. .. .. .. .. .. .. .. .. .. 147
  5. A mere Operator .. .. .. .. .. .. .. .. .. .. .. 148
  6. Ligature of both Carotids .. .. .. .. .. .. .. .. .. 149
  7. Ligature of the Gluteal Artery .. .. .. .. .. .. .. .. 150
  8. Operations on dilated Veins .. .. .. .. .. .. .. .. .. 150
  9. Dynamometer .. .. .. .. .. .. .. .. .. .. .. .. 150
  10. Mr. Crosse's Remarks on Medical Journals .. .. .. .. .. .. 151
2. Dr. Heygate on Tic Douloureux, Neuralgia, Fever, &c. .. .. .. .. .. 154
3. The British Medical Almanac .. .. .. .. .. .. .. .. .. .. 156
  1. Politics not proper in Almanacs .. .. .. .. .. .. .. .. 157
  2. Medical Statistics .. .. .. .. .. .. .. .. .. .. .. 157
4. Recent Works on Anatomy.
  1. The Cyclopædia of Anatomy, &c. .. .. .. .. .. .. .. .. 158
  2. Mr. Solly on the Human Brain .. .. .. .. .. .. .. .. 159
  3. Mr. Elkington's Description of the Skeleton .. .. .. .. .. .. 160
  4. Dr. Quain's Plates .. .. .. .. .. .. .. .. .. .. .. 160
  5. Messrs. Knox on the Varieties of Arteries .. .. .. .. .. .. .. 160

### Spirit of the English Periodicals, and Notices of English Medical Literature.

1. Dr. Symonds on Death.. .. .. .. .. .. .. .. .. .. .. .. 161

2. On Cerebro-spinal Irritation. By Mr. Cockburn .. .. .. .. .. .. 168

  Remarks by the Editors .. .. .. .. .. .. .. .. .. .. .. 169

3. Mr. Bloxam on Inversion of the Uterus .. .. .. .. .. .. .. .. 169

4. Spectral Illusions .. .. .. .. .. .. .. .. .. .. .. .. 173

5. Case of Spectral Illusions. By Mr. Craig .. .. .. .. .. .. .. 173

  Dr. Craigie's Comments on this Case.. .. .. .. .. .. .. .. .. 175

6. Wound and successful Ligature of the internal Jugular Vein. By Dr. Morgan .. 175

7. New kind of Truss for Prolapsus Uteri .. .. .. .. .. .. .. .. 177

8. New Mode of amputating the Penis. By Dr. Ogier .. .. .. .. .. .. .. .. .. 178
9. Modification of M. Dupuytren's Instrument for the Cure of Artificial Anus. By Dr. Lotz .. .. .. .. .. .. .. .. .. .. .. .. .. .. .. .. .. 179
10. Experiments and Observations on tying the Carotid and Vertebral Arteries, and the Pneumo-gastric and other Nerves, &c. &c. By Sir Astley Cooper, Bart. .. 180
11. Statistics of Remedies used in Dublin, and dispensed from Apothecaries' Hall there, from 1780 to 1836 .. .. .. .. .. .. .. .. .. .. .. .. .. 186
12. Abortion with tardy Expulsion of an undecomposed Fœtus. By Dr. Isaac Porter, 187
13. Observations on the Use of the Hydriodate of Potass as a Remedy in Venereal and Cachectic Complaints. By one of the Editors .. .. .. .. .. .. .. .. .. 188
14. Ulceration of the Cæcum, and Abscess of the Right Iliac Fossa, communicating with the Bladder, and producing symptoms of irritable Bladder. By Mr. H. Johnson 197
15. On Diseases connected with Spiculæ of Bone. By Dr. M'Cabe .. .. .. .. .. 200
16. Semi-poisoning by Iodine. By Dr. M'Cabe .. .. .. .. .. .. .. . . 201
17. Severe Injury of the Brain—Suppuration—Trephine—Incision of the Dura Mater, &c. By Mr. Forsayeth .. .. .. .. .. .. .. .. .. .. .. .. .. 202

### Spirit of the Foreign Periodicals, &c.

1. German Contributions to Practical Medicine. Communicated by Dr. Robert Thacker.
  1. State of Medicine in Chili. By Professor Poeppig . .. .. .. .. .. 203
  2. Case of Poisoning by Muriate of Barytes. Dr. Wach. .. .. .. .. 205
  3. Case of Tubal Pregnancy. By Dr. Dreger .. .. .. .. .. .. .. .. .. 206
  4. Case of Extra-Uterine Pregnancy occasioning Retroversion of the Uterus, 207
  5. Case of Pregnancy in which the Fœtus remained between Two and Three Years in the Abdomen.. .. .. .. .. .. .. .. .. .. .. .. .. . 208
  6. Proposal for removing Nævi Materni .. .. .. .. .. .. .. .. .. .. .. 209
2. State of the Medical Profession in France from the Revolution (1792) to the present time .. .. .. .. .. .. .. .. .. .. .. .. .. .. .. .. .. .. 210
3. Notice of some of the English, German, and Italian Medical Museums .. .. .. 213
Notice of Works received from Dr. Warburton, of Palermo .. .. .. .. .. .. 217
4. On the Chemical Properties of the Secretions in Health and in Disease, &c... .. 218
5. Researches on some of the Properties of Pus and of Blood .. .. .. .. .. 219
6. Extract from a Lecture by M. Serres on some Points in Organology .. .. .. 222
7. Examples of Double Uterus .. . .. .. .. .. .. .. .. .. .. .. .. .. 223
8. Treble Kidney in the Human Subject.. . .. . .. .. .. .. .. .. .. 224
9. Discussion at the Royal Academy on Apoplexy and Phrenology .. .. .. .. 225
10. Illustrations of various Lesions, &c. of the Nervous System .. .. .. .. .. 232
  *a.* Hysteria followed by Ischuria .. .. .. .. .. .. .. .. .. .. 232
  *b.* Case of Catalepsy .. .. .. .. .. .. .. .. .. .. .. .. .. .. .. 233
  *c.* Epilepsy of 33 years' standing, cured by the Removal of a Carious Portion of the Cranium .. .. .. .. .. .. .. .. .. .. .. .. .. 234
  *d.* Epilepsy cured by Nitrate of Silver .. .. .. .. .. .. .. .. .. 234
  *e.* Case of protracted Neuralgia of the Spermatic Nerves.. .. .. .. .. 234
  *f.* Neuralgia .. .. .. .. .. .. .. .. .. .. .. .. .. .. .. .. .. 235
  *g.* Curious Case of Metastasis of Neuralgia .. .. .. .. .. .. .. .. 236
  *h.* Nux Vomica in Treatment of Incontinence of Urine, &c. .. .. .. .. .. 236
  *i.* Medical Uses of Electricity .. .. .. .. .. .. .. .. .. .. .. .. 237
  *k.* Post-mortem Appearances in Cattle killed by Lightning .. .. .. .. 238
11. Poisoning from Colchicum .. .. .. .. .. .. .. .. .. .. .. .. .. .. 239

### Clinical Review, and Hospital Reports.

GUY'S HOSPITAL.

1. Guy's Hospital Reports. Nos. II. and III. for April and September, 1836 .. .. 241

   1. Observations on the Nature and Treatment of Ganglion, Bunion, &c. By Mr. Aston Key .. .. .. .. .. .. .. .. .. .. .. .. .. . .. 241

      a. Enlarged Bursa over the Patella .. .. .. .. .. .. .. .. .. 242

      b. Distortion of the Foot from Pressure .. .. .. .. .. .. .. .. 244

      c. Bunion.. .. .. .. .. .. .. .. .. .. . .. .. .. .. .. 245

NOTTINGHAM GENERAL HOSPITAL.

2. Spasms of the Muscles of the Neck .. .. .. .. .. .. .. .. .. .. .. .. .. 247
3. On several Affections of the Liver .. .. .. .. .. .. .. .. .. .. .. ..
   a. Observations on Fatty Degenerations of the Liver. By Dr. Addison .. 248
4. Observations on Jaundice. By Dr. Bright.. .. .. .. .. .. .. .. .. .. .. 251
5. Case of Cystine or Cystic Oxide Calculus. By Mr. Golding Bird .. .. .. .. 256
6. Remarkable Malposition of the Stomach .. .. .. .. .. .. .. .. .. .. .. 259
7. Statistics of Hospitals .. .. .. .. .. .. .. .. .. .. .. .. .. .. .. .. .. 261
   a. Number of Patients and of Deaths in the Hospitals of Great Britain . .. 262
   b. Advantages of due Statistical Returns from Hospitals.. .. .. .. .. .. 263
   c. Exaggerations of Number of Patients .. .. .. .. .. .. .. .. .. .. 264

ABERDEEN LUNATIC ASYLUM.

8. Dr. Macrobin's Report.. .. .. .. .. .. .. .. .. .. .. .. .. .. .. .. .. 265
9. Medical Statistics of the Army .. .. .. .. .. .. .. .. .. .. .. .. .. .. 269

NORTH LONDON HOSPITAL.

10. Recent Operations of removing the Superior Maxillary Bone .. .. .. .. .. 270

### Miscellanies.

1. Hahnemania .. .. .. .. .. .. .. .. .. .. .. .. .. . .. .. .. .. 277

2. How to make Stays . . .. .. .. .. . .. .. .. .. .. .. .. .. .. .. 280

3. Aneurism of the Arteria Innominata. By Sir D. Dickson, M.D. .. .. .. .. .. 282

4. Extract from the Charter of the London University . .. .. .. .. .. .. .. .. 282

5. Ordinance of the College of Surgeons on the Recognition of Teachers .. .. .. 285

6. Parisian Medical Club .. .. .. .. .. .. .. .. .. .. .. .. .. .. .. .. .. .. 286

7. Caries of the Bones of the Elbow-joint, cured by Excision .. .. .. .. .. .. 286

8. How to become a Crack Physician 150 Years ago. By Dr. Mandeville .. .. .. 287

9. Provincial Medical Journal .. .. .. .. .. .. .. .. .. .. .. .. .. .. .. .. 288

10. Leech Trade .. .. .. .. .. .. .. .. .. .. .. .. .. .. .. .. .. .. .. .. 288

11. A notable Discovery.... .. .. .. .. .. .. .. .. .. .. .. .. .. .. .. .. 288

12. Dr. Bostock's Erratum.. .. .. .. .. .. .. .. .. .. .. .. .. .. .. .. . 289

13. Phlebitis, Mr. Harrold .. .. .. .. .. .. .. .. .. .. .. .. .. .. .. .. .. 289

14. Obituary of Mr. Sheppard .. .. .. .. .. .. .. .. .. .. .. .. .. .. .. .. 289

BIBLIOGRAPHICAL RECORD .. .. .. .. .. .. .. .. .. .. .. .. .. .. .. .. .. 289

### Extra-Limites.

Dr. HAMILTON'S Reclamation .. .. .. .. .. .. .. .. .. .. .. .. .. .. .. .. 292

